# Acute Multi-Organ Toxicity During 24-Hour Dosing of Intravenous Amiodarone: A Case Report

**DOI:** 10.7759/cureus.25028

**Published:** 2022-05-15

**Authors:** Terrin Liwag, Kindchia Wong, Eladio Martinez, Steven Nguyen

**Affiliations:** 1 Internal Medicine, Western University of Health Sciences, Pomona, USA; 2 Family Medicine, Western University of Health Sciences, Pomona, USA; 3 Family Medicine, Desert Regional Medical Center, Palm Springs, USA

**Keywords:** side effects of amiodarone, drug reaction, drug-induced hepatitis, drug toxicity, acute kidney injury, hepatic toxicity, amiodarone

## Abstract

We present a unique case of a 60-year-old male with congestive heart failure who was admitted for a pre-syncopal episode and found to be in atrial fibrillation with rapid ventricular response (RVR). In order to effectively rate control the patient, he was administered an amiodarone bolus and intravenous (IV) infusion over 24 hours, along with a single oral 200 mg dose the following day. The patient subsequently developed acute hepatotoxicity along with features of acute kidney injury (AKI), pulmonary distress, and leukocytosis. After ruling out other etiologies for acute liver, pulmonary, and kidney injury, amiodarone-induced multi-organ toxicity was suspected and amiodarone was discontinued. Within hours of amiodarone discontinuation, the patient’s clinical status and organ function improved remarkably. In the setting of a patient being treated with IV amiodarone and presenting with sudden signs of dyspnea, acute elevation of transaminases and AKI within one to two days of initial dosing, acute amiodarone-induced organ toxicity should be considered.

## Introduction

Amiodarone is a widely used medication used to treat various types of arrhythmias including atrial fibrillation. While effective, amiodarone also accounts for 1-3% of all drug-induced liver injuries, most commonly in older patients with lower body surface area and dyslipidemia [1]. Approximately 25% of patients taking amiodarone develop asymptomatic increases in their serum aminotransferase levels due to tissue accumulation of amiodarone with long-term therapy [2]. However, a series of case reports over the past several decades have shown that the incidence of acute liver injury secondary to IV amiodarone is incredibly rare (<0.01%) [3]. Here we describe a case of a 60-year-old man with a past medical history significant for atrial fibrillation with rapid ventricular response (RVR) presenting with a pre-syncopal episode who was administered an amiodarone bolus and IV infusion over 24 hours, along with one oral 200 mg dose the following day. Following 24 hours of IV amiodarone administration, there was an insidious development of hepatotoxicity, acute kidney injury (AKI), and pulmonary distress. In addition, our patient developed an inflammatory response that manifested in a pneumonia-like presentation.

## Case presentation

A 60-year-old man presented to our emergency department (ED) via ambulance with a c-collar in place for neck pain and facial bruising status-post ground level mechanical fall and pre-syncopal episode. Paramedics stated the patient was found pale, cool, diaphoretic, hypotensive, and in atrial fibrillation with RVR. The patient admitted to feeling dizzy prior to his fall and endorsed cervical neck pain and facial pain which was worse upon palpation at the time of admission. He denied any loss of consciousness, severe headache, chest pain, shortness of breath, or abdominal pain prior to a near syncopal fall. He denied any numbness, tingling, ethanol alcohol (EtOH) use, or any other injury during his evaluation in the ED.

This patient’s past medical history is significant for congestive heart failure, type II diabetes, atrial fibrillation with RVR, one prior episode of near-syncope, and current methamphetamine and cannabis abuse. He was diagnosed with atrial fibrillation in 2019, currently managed on digoxin 125 mg daily. He denied any previous history of direct current cardioversion or ablation. The patient denied any history of tobacco or alcohol use. His other home medications included clopidogrel 75 mg daily, trazodone 50 mg at bedtime, spironolactone 50 mg twice a day, apixaban 5 mg twice a day, atorvastatin 40 mg daily, carvedilol 3.125 mg twice a day, enalapril 2.5 mg daily, nystatin 100,000 units/mL, semaglutide 2 mg/1.5 mg subcutaneous solution daily and empagliflozin 10 mg daily. He denied any previous history of liver dysfunction. 

Upon arrival at the ED, the patient was noted to be in atrial fibrillation with RVR. His blood pressure, temperature, and respiration rate were stable with oxygen saturation (spO2) of greater than 95% on room air. His physical exam was significant for tachycardia with an irregularly irregular pulse and 2+ pitting edema in the lower extremities bilaterally. Other system examinations were unremarkable. The patient’s significant laboratory findings and trends throughout the hospital course are detailed in Table 1.

**Table 1:** Summary of laboratory investigations from day one to day ten. THC: tetrahydrocannabinol; PCR: polymerase chain reaction; COVID-19: coronavirus disease 2019; MB: myocardial band

Laboratory Tests	Day 1	Day 2	Day 3	Day 4	Day 5	Day 6	Day 7	Day 8	Day 9	Day 10	Reference Values
White blood cells	5.5	7.1	11.1	8.8	7.5	4.2	4.4		7.1	6.4	4.2 - 10.8 × 10^3^/uL
Red blood cells	4.960	4.880	5.700	4.330	4.040	3.870	3.810		3.830	3.720	4.20 - 5.80 × 10^6^/uL
Hemoglobin	14.7	14.6	16.6	12.8	12.2	11.3	11.4		11.4	11.1	13.5 - 17.0 g/dL
Hematocrit	44.4	44.1	50.4	38.6	36.0	33.8	33.9		33.6	32.9	38.0 - 50.0%
Mean Corpuscular Volume	89.5	90.4	88.3	89.1	89.0	87.4	89.1		87.6	88.2	80 - 100 fL
Thyroid Stimulating Hormone		3.18							4.36		0.4 - 4 mI U/L
Procalcitonin				1.540		0.718					<0.1 ng/mL
Creatine kinase-MB		24.8									5 - 25 IU/L
Troponin I	0.559	27.70		3.870	3.320						0 - 0.04 ng/mL
Troponin T		1596									<14 ng/L
Total Bilirubin	1.6	1.4	3.2	2.3	2.2		2.0	1.4		1.2	0.2 - 1.3 mg/dL
Alkaline phosphatase	128	93	120	73	89		86	133	122	106	38 - 126 Iu/L
Alanine transaminase	49.0	44.0	2,933.0	1,870.0	1,390.0	1,063.0	887.0	710.0	539.0	400.0	10 - 60 Iu/L
Aspartate aminotransferase	122.0	124.0	8,143	3,191.0	1,478.0	821.0	711.0	351.0	187.0	112.0	10 - 42 Iu/L
Blood urea nitrogen	34.0	31.0	39.0	64.0	65.0	54.0	38.0	23.0	22.0	24.0	5 - 25 mg/dL
Creatinine	1.4	1.2	2.1	2.4	1.9	1.5	1.2	1.1	1.1	1.2	0.61 - 1.24 mg/dL
Glomerular Filtration Rate		>60.0	32.4	27.8		47.7	>60.0	>60.0	>60.0	>60.0	70 - 99 mg/dL
Prothrombin time	13.5			23.5		15.1					11.0-12.5 seconds
International normalized ratio	1.3			2.3		1.5					<1.1
Partial thromboplastin time	26.7	55.0	44.7								60-70 seconds
Amphetamine urine screen	Positive										N/A
Cannabis urine screen	Positive										N/A
THC urine screen	Positive										N/A
Amphetamine urine screen	Positive										N/A
COVID-19 PCR test	Negative			Negative	Negative						N/A
Influenza A antigen test					Negative						N/A
Influenza B antigen test					Negative						N/A

**Figure 1:**
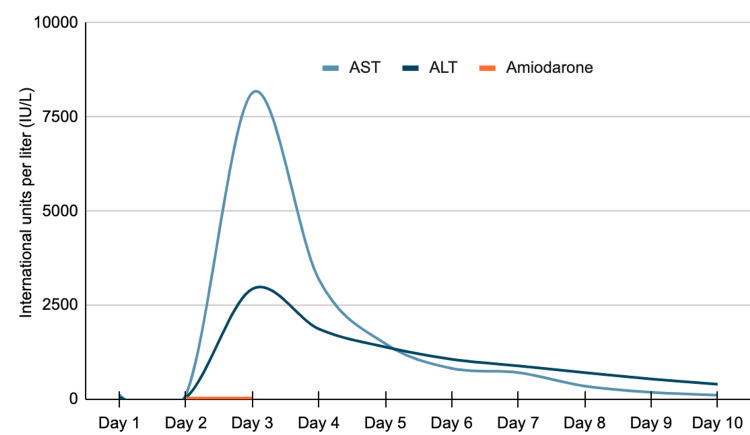
Transaminase (AST/ALT) levels over a 10-day hospital course, correlated with amiodarone dosing. AST: aspartate aminotransferase; ALT: alanine aminotransferase

Of note, the toxicology screen was positive for amphetamine, cannabis, methamphetamine, and tetrahydrocannabinol (THC). His blood alcohol level was <10. On day one of hospital admission, labs showed white blood cell (WBC) of 5.5 (reference values 4.2 - 10.8 K/uL), troponin 0.559 (reference value < 0.04 ng/mL), aspartate transaminase (AST) 122 (reference values 10 - 42 IU/L), alanine transaminase (ALT) 49 (reference values 10 - 60 IU/L), international normalized ratio (INR) 1.3, creatinine1.4 mg/dL (reference values 0.44 - 1.0 mg/dL), and blood urea nitrogen (BUN) 34.0 mg/d (reference values 5 - 25 mg/dL) (Figure 1).

**Figure 2:**
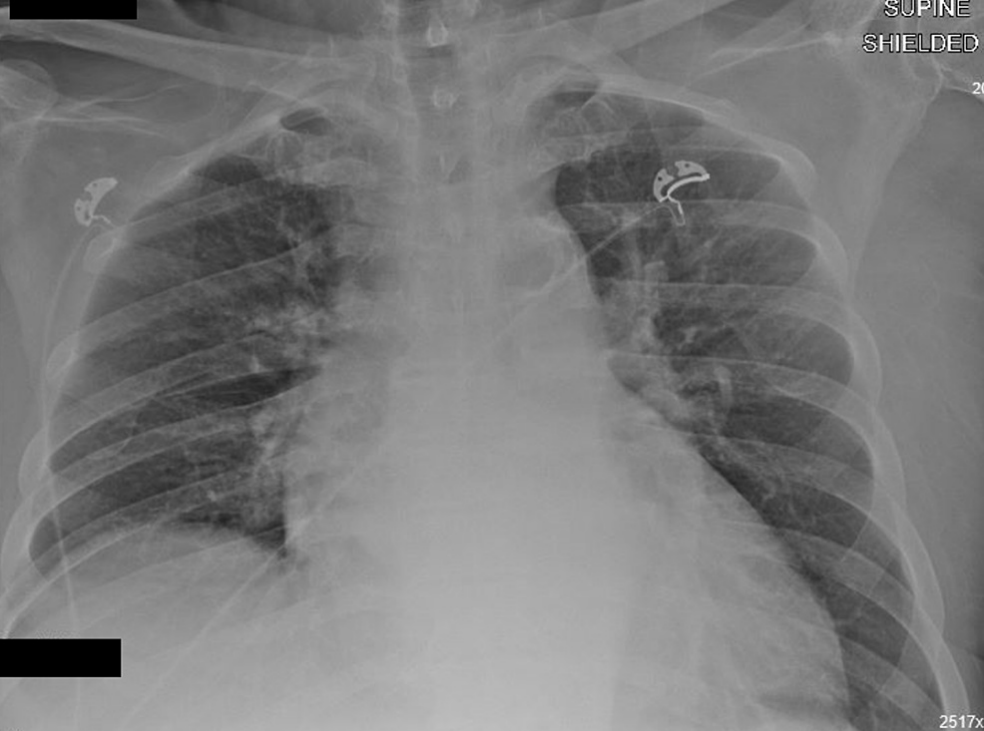
Chest x-ray on day one does not demonstrate any acute airspace opacities.

The CT head, CT cervical spine, chest x-ray, and pelvis x-ray were all unremarkable for any acute fractures or traumatic malalignment. The chest x-ray on admission did not demonstrate any acute airspace opacities (Figure 2).

**Figure 3:**
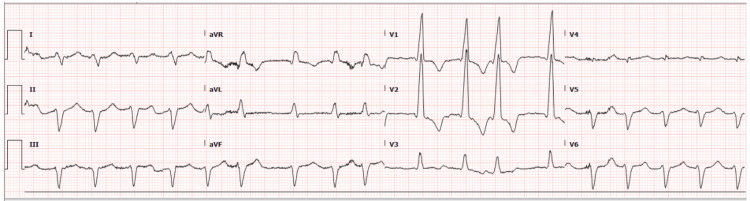
ECG on day three showed atrial fibrillation with RVR, extreme right axis deviation, intraventricular conduction delay, and a possible anteroseptal myocardial infarction of indeterminate age. ECG: electrocardiogram; RVR: rapid ventricular response

An echocardiogram demonstrated a dilated left ventricle, severely reduced left ventricular systolic function, and an ejection fraction estimated in the range of 15-25%. There was evidence of global hypokinesis, mild mitral annular calcification, and mild thickening of aortic valve leaflets. The diastolic function could not be assessed. ECG demonstrated atrial fibrillation with RVR, extreme right axis deviation, intraventricular conduction delay, and a possible anteroseptal myocardial infarction of indeterminate age (Figure 3).

**Figure 4:**
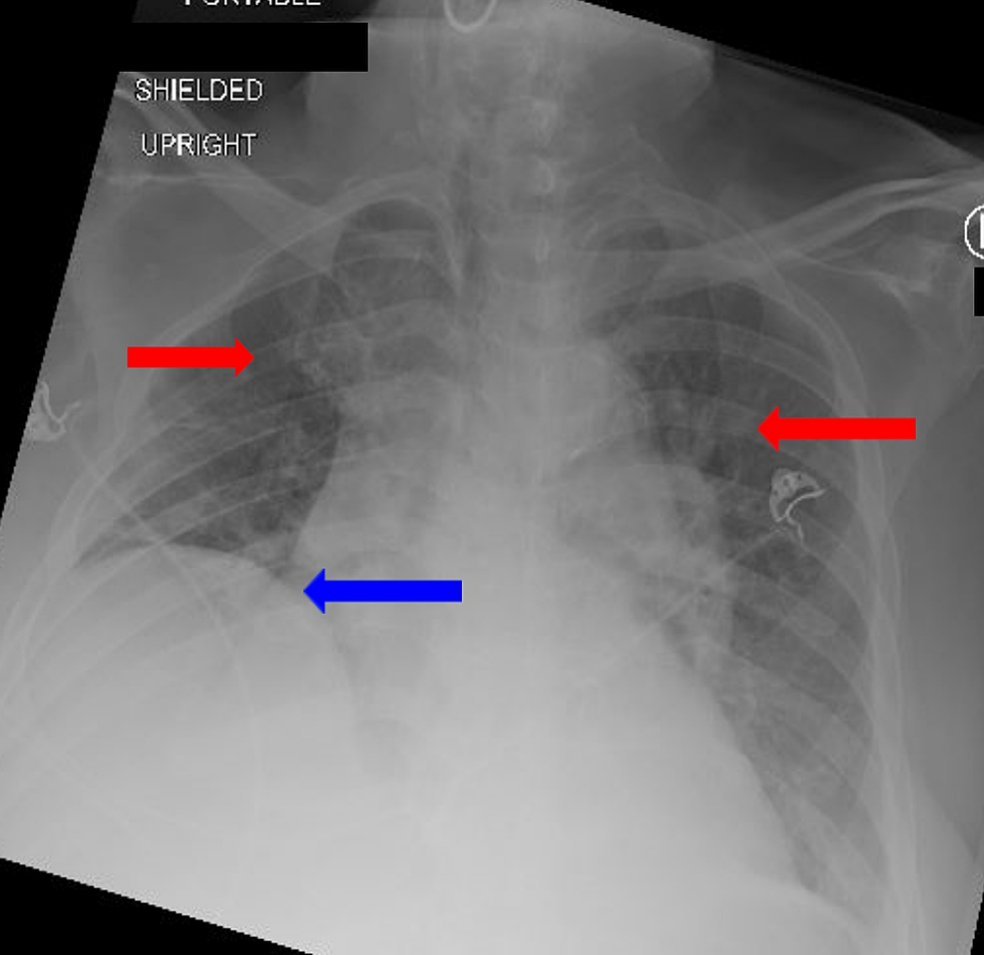
Chest x-ray on day three demonstrated pulmonary vascular congestion (red arrows) and right hemidiaphragm elevation (blue arrows). No consolation or lobar opacities were visualized.

He was initially given 20 mg of oral diltiazem and a subsequent diltiazem IV infusion, with no improvement to his heart rate. At 17 hours after admission, he was placed on amiodarone 150 mg IV bolus over 10 minutes, followed by 360 mg over six hours (0.1mg/min), followed by 540 mg over the remaining 18 hours (0.5 mg/min) for a total of 1050 mg over 24 hours. Along with the amiodarone infusion, he also received a total of carvedilol 3.125 mg orally twice a day, rosuvastatin 20 mg oral daily, magnesium sulfate 2 g IV solution, insulin lispro sliding scale, and Furosemide 40 mg Inj IV twice daily within the first 24 hours of admission. On day three, the patient was switched from IV amiodarone to oral amiodarone 200 mg twice a day. At this time, the patient's liver enzymes demonstrated a drastic increase, with an ALT of 3,353.0, AST >7,000, and partial thromboplastin time (PTT) of 44.7. A decline in renal function was also noted as creatinine became elevated to 2.1 mg/dL, and BUN 39.0 mg/d. Simultaneously, the patient began to show signs of possible pneumonia as he demonstrated leukocytosis (WBC 11.3), increased respiration rate of 22 breaths/min, spO2 of 68% on 15 L/min non-rebreather, heart rate of 124 bpm, temperature of 37.6 C, and blood pressure of 94/55 mmHg. From the deterioration of his clinical status and the deranged laboratory values, the diagnosis of fulminant hepatitis was made. In addition, he was also started on intravenous ceftriaxone 1g daily and doxycycline 100mg twice daily, and blood cultures were ordered for sepsis due to presumed community-acquired pneumonia. One hour following the patient's hypotensive state, his blood pressure stabilized without the need for any intervention. A repeat chest x-ray done on day three demonstrated pulmonary vascular congestion and right hemidiaphragm elevation (Figure 4).

The patient was retested for coronavirus disease 2019 (COVID-19) and influenza A and B, which were once again negative. Per medication history and chart review, he was not recently prescribed or exposed to hepatotoxic medications. Other causes of hepatic enzyme elevation were investigated by hepatic ultrasonography which demonstrated cirrhosis with mild ascites. Tests for specific viral etiology including a hepatitis B surface antigen, hepatitis B with corrected IgM, hepatitis A IgM, and hepatitis C antibody screen were found to be negative and blood cultures returned negative for bacterial infection. 

Given the close timing between amiodarone initiation and the patient’s abrupt decline in liver, lung, and renal function, amiodarone toxicity was considered the potential cause of the patient’s symptoms. The Naranjo adverse reaction score was seven (previous conclusive reports on this reaction +1, adverse event appeared after the suspected drug was administered +2, adverse event improved when the drug was discontinued or a specific antagonist was administered +1, there were no alternative causes that could on their own have caused the reaction +2, did the reaction reappear when placebo was given 0, was the drug detected in blood or other fluids in toxic concentrations 0, reaction was more severe when the dose was increased or less severe when the dose was decreased 0, similar reactions to similar drugs in previous exposure 0, adverse event was confirmed by objective evidence +1). According to the Naranjo algorithm, a score of 7 suggests amiodarone was a probable cause for acute liver toxicity [4]. Amiodarone was subsequently discontinued and the patient was started on digoxin 60 mcg orally daily. After amiodarone discontinuation, the patient’s clinical presentation significantly improved as he was downgraded from 15L non-rebreather to >95% spO2 on room air by day five. On day eight, his ALT was 710 and AST 351. Renal function improved with a creatinine of 1.1 mg/dL and BUN 23.0 mg/d. 

Despite the improvement of this patient’s liver and kidney function, the remainder of his hospital course was complicated by a duodenal ulcer on day eight confirmed by esophagogastroduodenoscopy (EGD). EGD did not demonstrate any gastric varices or portal hypertensive gastropathy. The ulcer was cauterized and a repeat EGD showed complete resolution of the bleed. The patient then underwent cardiac catheterization on day nine to further evaluate the cause of his presyncopal episode for which he was found to have multi-vessel coronary artery disease. The patient was transferred to a tertiary care facility for further evaluation of a possible coronary artery bypass graft. At this point, no further follow-up was performed.

## Discussion

Amiodarone is an iodine-containing antiarrhythmic drug that is metabolized in the liver to mono-N-desethylamiodarone (DEA) by cytochrome P-450 enzymes. Amiodarone is known for its lipophilic property and its ability to perfuse through adipose-dense organs like the lungs, liver, spleen, and skin where it ultimately accumulates [5]. However, the most commonly acknowledged toxicities associated with amiodarone are through adipose tissue deposition via long-term oral use of the antiarrhythmic drug. The pathophysiological mechanism behind acute hepatitis following IV administration of amiodarone, as seen in our patient, is still unclear and extremely rare. Among the limited documented cases of this phenomenon, Rhodes et al. theorized that the acute amiodarone-induced hepatitis was due to an emulsifying component found in IV amiodarone called polysorbate-80. A similar reaction was observed among premature infants following the use of E-ferol, an IV formulation of Vitamin E containing polysorbate-80 and polysorbate-20 [6]. E-ferol Syndrome, a constellation of liver, pulmonary, and renal failure, was observed in premature infants following exposure to polysorbate-80. The symptoms seen in this syndrome manifested similarly to our patient, causing us to suspect an analogous pathophysiological mechanism.

Whereas the most common adverse reaction to amiodarone is pulmonary toxicity, additional multi-organ involvement has been observed. In the case of our patient, there was an insidious development of hepatotoxicity, AKI, and pulmonary distress. Following 24 hours of IV amiodarone administration, our patient developed an inflammatory response that manifested in a pneumonia-like presentation. Despite our patient meeting systemic inflammatory response syndrome (SIRS) criteria based on multiple abnormal vital signs and mild leukocytosis, there was no definitive infection to explain the onset of symptoms. Two chest x-rays obtained during the hospital stay, one at admission and another on day three, remained unremarkable for occult infection. Given that the onset and resolution of the pneumonia-like and hepatic symptoms coincided with the initiation and cessation of the IV amiodarone, there is high clinical suspicion that the inflammatory response was attributed to the amiodarone as opposed to sepsis. Blood cultures and viral panels yielded no definitive source of infection. This aligns with another documented instance wherein a multi-systemic inflammatory response was observed in a patient following IV amiodarone supporting this hypothesis [7]. In addition, this patient was found to have underlying cirrhosis as demonstrated on a liver ultrasound only after amiodarone was discontinued. Patients with cirrhosis may be at an increased risk of acute worsening of liver function, especially in the presence of hepatotoxic agents [8].

Although the acute development of AKI is likely to be attributed to amiodarone toxicity due to the close temporal relationship, it is important to consider other possible causes. Given the fact that this patient was taking rosuvastatin 20 mg orally every day in concordance with amiodarone, a CYP3A4 inhibitor, it is possible that a drug interaction occurred leading to slowed metabolism of rosuvastatin. A known result of this drug interaction is rhabdomyolysis leading to AKI [9]. At the time of this patient’s course, creatine kinase (CK) levels were not obtained as the medical team did not have high clinical suspicion that rhabdomyolysis could be a possible cause of AKI within this clinical scenario. Nevertheless, we believe it is important to recognize that rhabdomyolysis could be an important complication in patients taking amiodarone with a concomitant statin presenting with elevated BUN and creatinine levels. 

We have also considered the possibility that acute transaminitis and liver failure in our patient could have been due to ischemic hepatitis [10]. Ischemic hepatitis can occur in the setting of hemodynamic instability, resulting in ischemic injury to hepatocytes. This can present as a rapid increase in serum aminotransferase levels following a hypotensive or hypoxic insult. Our patient presented to the ED with hypotension and cardiac arrhythmia. His hospital course was further complicated by acute respiratory distress and an upper GI bleed from a duodenal ulcer. Although his liver enzymes and renal functions normalized shortly after the discontinuation of amiodarone, we cannot dismiss the possibility of ischemic hepatitis given his presentation of atrial fibrillation, decreased cardiac function secondary to multi-vessel coronary artery disease, development of acute respiratory distress, and volume loss via an upper GI bleed. However, we recognize that in the setting of an obvious hepatotoxic agent like amiodarone, suspicions of ischemic hepatitis are low. In addition, we do not have knowledge of his baseline cardiac function prior to admission to compare. 

In hindsight, liver function and coagulation tests should have been performed more frequently in this patient after the initial rise in serum aminotransferases was observed to indicate the peak value of liver enzymes. In addition, although hepatic toxicity became evident during the 24-hour IV amiodarone dosing period, the choice to discontinue amiodarone was not made until the next day as other possible sources of multi-organ damage were being considered. Perhaps closer monitoring of labs and prompt cessation of amiodarone may have improved this patient’s clinical outcome at an increased rate. Finally, in scenarios where patients present with acute liver decompensation with an unknown cause, etiologies such as autoimmune hepatitis should be considered and evaluated.

## Conclusions

This case report supports current literature suggesting that amiodarone has the ability to cause fulminant multi-organ toxicity over a short period of time. Patients given IV amiodarone should be monitored closely for signs of hepatotoxicity, renal toxicity, pulmonary toxicity, and acute decompensation in clinical status. Laboratory testing should include thyroid, renal, and liver function panels along with close monitoring of respiratory status. If transaminitis or AKI becomes evident, as seen in this case, amiodarone should be discontinued promptly and a full workup for other possible causes should be performed concurrently.
